# Humeral Head Replacement in the Treatment of Comminuted Proximal Humeral Fracture

**DOI:** 10.1111/os.12732

**Published:** 2021-01-05

**Authors:** Xinghuo Zhang, Yakui Zhang, Tao Guo, Liang Liu, Wenhao Cheng

**Affiliations:** ^1^ Department of Bone and Joint Surgery and Sports Medicine of Beijing Luhe Hospital Capital Medical University Beijing China

**Keywords:** Humeral head, Proximal humeral fracture, Replacement

## Abstract

**Objective:**

To investigate the outcomes of humeral head replacement in the treatment of patients with comminuted proximal humeral fracture.

**Methods:**

Between February 2013 and September 2016, 56 patients underwent humeral head replacement in our hospital. Of them, 18 cases were diagnosed as comminuted proximal humeral fracture before the operation. The mean age of the patients was 69.5 years old (ranging from 61 to 79 years old). Of them, there were six males and 12 females. All the patients in this group had fresh fractures. They were all treated by artificial humeral head replacements. After the prosthesis was fixed by bone cement reliably, the greater or lesser trochanter and prosthesis handle were sutured and fixed firmly. The interval time from injury to operation ranged from 1 to 5 days. The Constant Functional Score, operation time, blood loss, nerve injury, joint dislocation rate, and infection rate were recorded at the final follow‐up. The clinical data of these patients were retrospectively studied. All of the data were recorded in average form.

**Results:**

In this study, the mean duration of follow‐up was 4 years, ranging from 3 to 6 years. The operation time ranged from 75 to 120 min, with the average of 82 min. The blood loss ranged from 100 to 400 mL, with the average of 210 mL. The mean score of Constant Functional Score was 83.5 ± 3.1. Of them, 14 cases achieved excellent and good (scores of more than 80), and four cases achieved moderate and poor (scores of less than 80). No patient suffered from joint dislocation, unstable joint, or infection after the operation. There were two patients with axillary nerve injury before the operation. However, the function could be recovered within 3–6 weeks after the surgery.

**Conclusion:**

The artificial humeral head replacement could be applied for the treatment of patients with comminuted proximal humeral fracture. During the surgery process, the stable structure of shoulder joint could be completely restructured, and the rehabilitation plan should be adjusted reasonably and timely after the operation.

## Introduction

Proximal humeral fracture accounted for 4%–5% of patients with fracture^1^, ^2^, ^3^. With the aging of the population in China, the incidence of proximal humeral fracture has been gradually increasing year by year in elderly patients whose ages are more than 65 years^2^. Due to the osteoporosis of older patients, the incidence of Neer three or four parts fractures has increased in recent years.^1^, ^4^, ^5^. Complications such as nerve injuries, soft tissues contusions, rotator cuff tears enhanced the difficulties of the surgeries treatment in these kinds of fracture. Van de Water mentioned that a proximal humeral fracture is debilitating for the person directly after the trauma with loss of arm function and severe pain, and often results in ongoing disability with a prolonged period of recovery and rehabilitation^22^.

For the treatment of Neer three or four parts proximal humeral fracture, only conservative treatment methods are used in older patients who cannot bear the surgical procedures due to serious medical complications and chronic diseases^4^, ^6^. Many kinds of surgical treatment methods were applied in clinical practice. Of them, open reduction and internal fixation with screws and plates, and artificial shoulder prosthesis replacements, were wide spread used all over the world.

Open reduction and internal fixation with screws and plates was effective in the treatment of simple fractures of the proximal humeral fracture^5^, ^7^. This method could retain the bone mass to the maximum degree, and the side effect for the shoulder joint was low. However, for patients with comminuted fractures of proximal humeral fracture, after the treatment of traditional open reduction and internal fixation or intramedullary nailing, the necrosis rate is higher than other techniques, and the clinical outcomes are not relatively good^3^, ^4^, ^5^. For most of the patients, the necrosis rate is related to the reduction techniques. Kloub found in the group of excellent reduction, mean relative Constant Score (CS [rel]) was 88% and the rate of necrosis was 2%. Moderate reduction quality deteriorated CS (rel) to 70% and head necrosis rate rose up to 28%. If reduction was poor, mean CS (rel) was 52% and the rate of complete necrosis rose to 60%^5^. Greiner followed 48 patients with proximal humeral fractures treated by open reduction and angular stable plate fixation, and found long‐term complications need to be evaluated further^4^.

In recent years, the technical improvement of artificial shoulder prosthesis replacement make the humeral head replacement applied for the treatment of complicated proximal humeral fracture had achieved satisfactory clinical efficacy^3^, ^6^. For most of the patients with proximal humeral fractures, the humeral head replacement is still an effective method. Some authors found this method should be paid more attention in some particular circumstances. Bishop reported that glenoid loosening and the subsequent possibility of a difficult revision with bony deficits have led many to favor humeral head replacement alone^9^. Sebastia‐Forcada also mentioned that revision from humeral head replacement to reverse shoulder arthroplasty appear to improve outcomes^10^. Greiwe retrospectively analyzed 30 patients with fractures of the proximal humerus, and found that after treatment of humeral head replacement, the head split fractures of the proximal humerus got more forward elevation ability than the control group^6^. But the clinical outcomes of humeral head replacement in the treatment of comminuted proximal humeral fractures was still not paid enough attention in former studies. We chose those patients with comminuted proximal humeral fractures treated with the humeral head replacement in our center to find the outcomes of medium‐term follow‐up.

## Methods

Between February 2013 and September 2016, 56 patients with complicated proximal humeral fracture had received humeral head replacement in our hospital. Among them, 18 cases have been diagnosed as comminuted proximal humeral fracture before operation. The data was reported and listed as below.

### *General Data*

Inclusion criteria: (i) diagnosed as comminuted proximal humeral fracture; (ii) treated with humeral head replacement without other treatment methods; and (iii) retrospective analysis.

Exclusion criteria: (i) combined with fractures of other parts of the same side upper limb; and (ii) combined with severe medical disease that could not bear the surgical intervention.

Of all 18 cases, there were six males and 12 females, with an average age of 68.5 years (range, 61 to 79 years). Of them, seven cases suffered from traffic injury, and 11 cases suffered from fall damage. All patients of this group had fresh fracture. According to Neer classification, 18 patients had comminuted fracture (three or four parts fracture). Of them, two patients were combined with injury of axillary nerve. All patients underwent artificial humeral head replacement within 1–5 days after the injury.

### *Treatment Strategy*

According to the results of preoperative affected side X‐ray films and computed tomography (CT) results, the conditions of fracture could be evaluated comprehensively.

#### *Anesthesia and Approach*

The patient underwent general anesthesia and was required to keep the beach chair posture. The approach of pectoralis‐deltoid was made. The incision originated from the middle‐outside third of the clavicle, and extended to the proximal humerus towards inferior outside. Through the interval between the deltoid muscle and the pectoralis major muscle, the cephalic vein was exposed and separated. Then the cephalic vein was pulled towards outside. When the cephalic vein was exposed completely, the attachment part on the humerus of the musculus subscapularis and pectoralis major muscle could be cut and marked. And after the surgery, the suture and fixation were completed.

The broken ends of the fractured bone could be revealed layer by layer. When the patient was combined with the avulsion fracture of greater trochanter, and the attaching rotator cuff tissue should be marked by suture. The humeral head should be revealed clearly and successfully removed. The relationship between the humeral head and surrounding tissues should be clarified. The diameter of the removed humeral head should be measured in order to determine the model and type of the humeral head prosthesis. The cancellous bone in the humeral head should be removed and adjusted to the granulate state, in order to prepare for bone graft. The violent traction should definitely be avoided in order to prevent the damage of important vessels and nerves.

#### *Prostheses Fixation*

The scapula glenoid should be cleaned and explored, in order to confirm no fraction of scapula glenoid region. Then the test model file was used to expand the marrow in order to determine the appropriate size of the prosthesis handle. The distal medullary cavity plug should be placed at the medullary cavity of humerus. During the processes of prosthesis implantation, the prosthesis hypsokinesis should be kept between 20° to 30°. According to the deltoid muscle tension, the subacromial space was about 1 cm. The upper arm was pulled, then the movement of humeral head prosthesis (up and down) was observed. The following conditions, such as joint stability and no joint dislocation, should be determined and the height of the prosthesis should be adjusted. The cancellous bone granules of the humeral head were grafted inside the intervals between greater or lesser trochanter and prosthesis. Then, the greater or lesser trochanter and rotator cuff were carefully repaired. After the prosthesis was fixed by bone cement reliably, the reset greater or lesser trochanter and prosthesis handle should be sutured and fixed firmly (forward and backward directions). The greater or lesser trochanter and rotator cuff tissues underwent the ring ligation procedure through the longitudinal direction of humeral trunk fascia line, in order to ensure the suture and fixation reliability.

The motion of the joint should also be examined. All the patients received humeral head replacement, and bone cement prosthesis was applied. In the patients of this study, the long bicipital tendon was cut, which could effectively reduce the occurrence of postoperative pain. Then the joint capsule was sutured, the negative pressure drainage tube was implanted, and then the incision was closed layer by layer.

### *After Treatment*

The humeral shaft was protected by the brace for about 6 weeks after the operation. The affected limb was required to avoid internal rotation, in order to decrease the tension of rotator cuff tissue, and be beneficial to the union of greater or lesser trochanter. The patient was required to start the active function exercise of affected elbow, wrist, and hand at the next day after the operation in order to alleviate the postoperative affected limb swelling. The patient was required to start active activities when the results of X‐ray indicated that greater or lesser trochanter showed bone union about 3–4 weeks after the surgery.

### *Follow‐Up Methods*

The patients were required to be followed up every 2 weeks by the physical therapist, and were guided in the rehabilitation training. The patients were required to recheck the X‐ray at time points of 1, 2, 3, 6, and 12 months after the surgery (including X‐rays were taken at 1, 2, 3, 6, and 12 months after surgery including anteroposterior position, scapular lateral position, axillary position). The healing conditions of fractures were observed dynamically. Then, the patients were required to be followed up at least once every year.

### *Evaluation Methods*

The Constant Functional Score was used to evaluate postoperative recovery of shoulder function in adults. The Constant Functional Score system includes two parts. The subjective part included pain and daily life abilities (35 points). The objective part included shoulder function and muscular strength (65 points). The score standard had a maximum of 100 points (best function). A total score of less than 70 is considered as poor outcome, 70–80 is fair, 80–90 is good, and 90–100 is excellent^23^.

### *Statistical Methods*

All statistical analyses were conducted using the Statistical Package for Social Sciences (SPSS) 17.0 for Windows (SPSS Inc., Chicago, IL). All *P* values are two‐sided, with a significance level of 0.05. The result of statistical data were presented as mean ± SD.

## Results

### *General Results*

All 18 cases completed the follow‐up. The mean follow‐up time ranged from 3 to 6 years, with the average time of 4 years.

### *Operative Time*

The operation time was recorded during the operation, which was recorded from the time of incision making to the end of suture. It could reflect the period of the tissue exposed in the air, which has some relationship with the complications such as infection rate. The operation time ranged from 75 to 120 min, with the average time of 82 min.

### *Blood Loss*

The blood loss was recorded as the volume of intraoperative blood loss. It could reflect the damage of the tissues. The blood loss ranged from 100 to 400 mL, with the average of 210 mL.

### *Constant Functional Score*

The clinical evaluation was evaluated by Constant Functional Score. The average score of the final follow‐up was 83.5 ± 3.1, compared with 32.6 ± 4.2 preoperational. Of them, 14 cases achieved excellent and good (more than 80 scores), and four cases achieved moderate and poor (less than 80 scores). The rate of excellent and good effect was 77.8%.

### *Complications*

No patient occurred accessory nerve damage, infection, peri‐prosthetic fracture, joint instability after the treatment. However, two patients with the combination of dislocation and axillary nerve injury suffered shoulder subluxation within 3 months after the surgery, and the patients recovered gradually within 3 months after the surgery. The patients with axillary nerve injuries mainly performed humerus head oppression, traction, and individual adhesion. No case observed the symptom and sign of nerve rupture.

## Discussion

The shoulder joint is the most unstable joint in the body, which is easy to suffer fractures^2^, ^3^, ^7^. The size of humerus head was three to four times as large as glenoid cavity^3^. The stability of the joint was not only dependent on bone structure, but also joint capsule, ligaments, muscles and other soft tissues. Of these above‐mentioned structures, the most important structure was the rotator cuff tissue, which was attached to the greater or lesser trochanter and is easily injured during the femoral head fracture. Therefore, any method for the treatment of shoulder joint disease should find the balance between stability and activity.

In the treatment strategies of comminuted proximal humerus fracture, the common surgical methods could be divided into three categories, which include open reduction and internal fixation, artificial humeral head replacement, and total shoulder arthroplasty^1^, ^3^, ^7^. The results of long‐term follow‐up for total shoulder arthroplasty indicated that, compared with humeral head replacement, total shoulder arthroplasty could significantly alleviate shoulder joint pain, meanwhile, the range of motion could be retained with the greater degree in the region of the shoulder joint^10^. However, for most patients with comminuted proximal humeral fracture, the cartilage state in the region of scapula glenoid side was still good and the general degree of osteoporosis in the whole body was poor. The prosthesis in the region of scapula glenoid side suffered loosening, or the bony defect problem subsequently occurred. These questions limited this kind of surgery applied in the clinical promotion for the treatment range of proximal humeral fracture^10^, ^11^, ^21^. The open reduction and internal fixation could retain the bone mass to the maximum degree, but the occurrence rate of avascular necrosis in the region of femoral head was not low^8^, ^20^. Greiner *et al*. studied patients with proximal humeral fractures who underwent the open reduction and internal fixation surgery. The occurrence rate of avascular necrosis in the region of femoral head in end‐stage reached up to 18.75%^4^.

We believed that for patients with comminuted proximal humeral fracture, the surgery strategy still should focus on humeral head replacement. In our study, 14 cases achieved satisfactory curative effect. That indicated that this kind of classical surgery exerted better effect on the treatment of comminuted proximal humeral fracture.

**Fig. 1:**
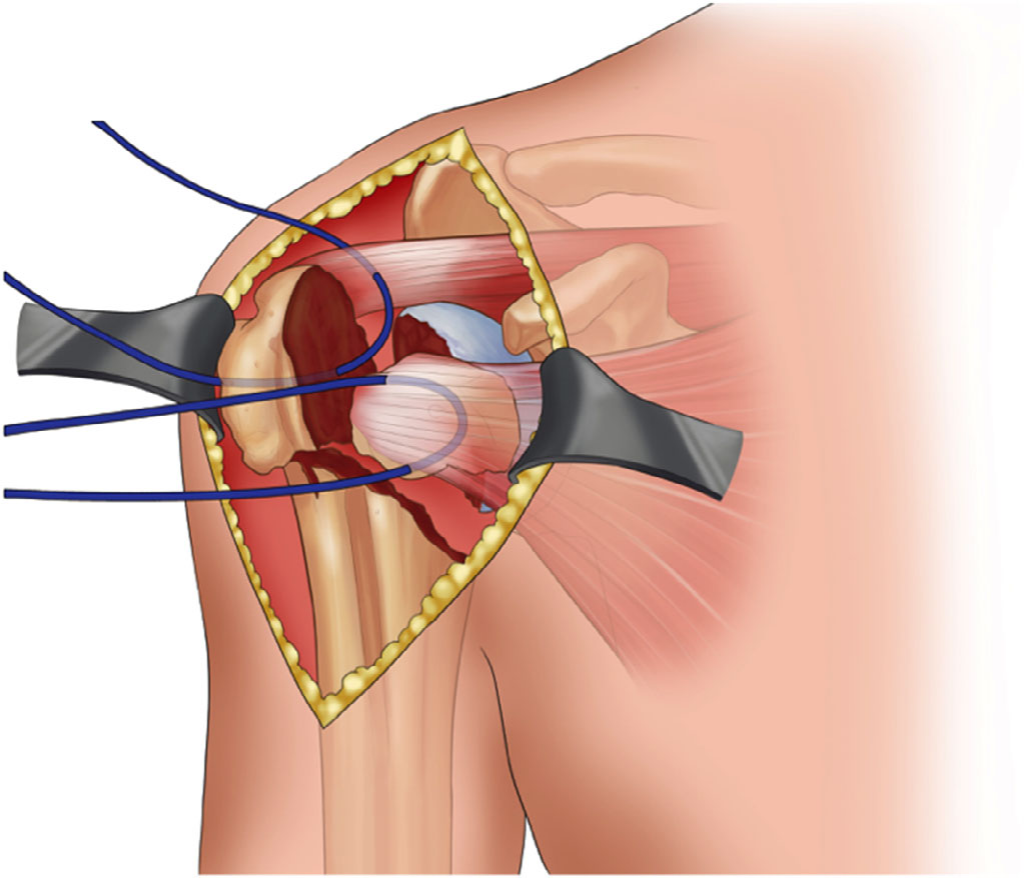
This figure demonstrated the way of protection of rotator cuff which attached the great and lesser trochanter during exploration. It is very important for later reconstruction after the implant fixation.

**Fig. 2:**
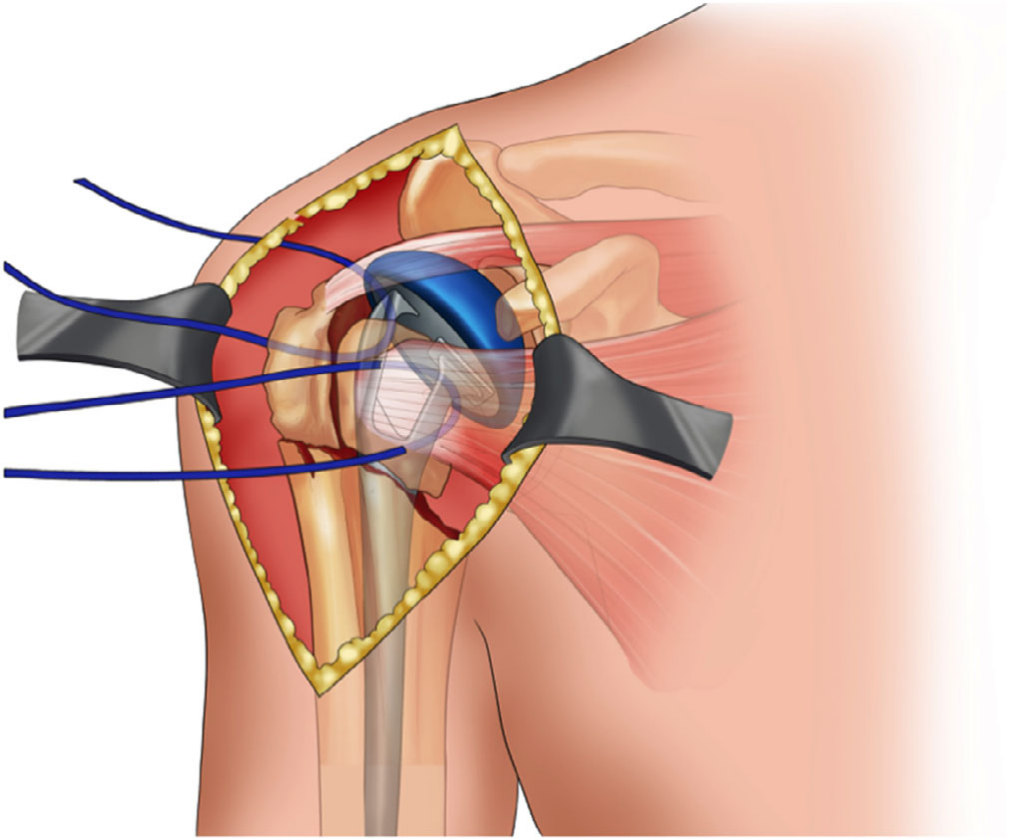
This figure showed the status of fixation of the implant. The key point is to keep the protheses at the position of retroversion between 20 and 30 degree.

The stability of the shoulder joint is the key for the pursuit of great shoulder range of motion (ROM). The role of rotator cuff played the greatest role, its integrity and restoration of spatial position was extremely crucial for the stability reconstruction of shoulder joint^12^, ^15^. In our study, although the rotator cuff showed the damage to varying degrees, the continuous relationship between rotator cuff and greater or lesser trochanter could still be retained. If the trochanter could be reconstructed, it should be fixed on the humerus by the steel wire as far as possible. If the trochanter could be reconstructed after smash, then the rotator cuff should be sutured and fixed on the prosthesis hole, and the bone graft should be implanted on the position of collum chirurgicum. We found that the anatomical replacement of greater trochanter was not only the important basis for the repair of the rotator cuff, but also the anatomical replacement of trochanters could clearly identify the intertubercular sulcus. Through the trochanters and intertubercular sulcus, it could accurately determine the normal angle of humeral head in this patient^13^, ^16^. However, even if the damaged rotator cuff should be fixed on the prosthesis, the postoperative lifting function of shoulder joint in two cases with rotator cuff tear were still limited (Fig. 1,2), which also indicated that the restoration of greater trochanter position played an important role in the function of the shoulder joint^13^, ^14^.

In addition, the tendon of the long head of biceps brachii exerted the crucial effect on the stability of the shoulder joint. In a randomized controlled prospective cohort study conducted by Soliman *et al*., the result showed that the Constant Functional Score of tendon of long head of biceps brachii in reconstruction group was 74.4 ± 6.5, which was higher than non‐reconstruction group (Constant Functional Score = 69.8 ± 6.6). The tendon of long head of biceps brachii could also be one of the factors which could influence the stability of shoulder joint^17^. In the patients of our group, seven patients suffered injuries of tendon of long head of biceps brachii, and underwent tenodesis operations. In addition, although 11 patients had injuries of tendon of long head of biceps brachii, they also underwent the process of tenotomy and fixation *in situ* after the reconstruction of greater or lesser trochanter. Through the above‐mentioned techniques, during the follow‐up period of the 18 patients in this study, 17 patients did not suffer dislocation of shoulder joint and other related complication, which basically ensured the stability of the shoulder joint. During the early postoperative period, one case still had shoulder subluxation. This patient was combined with axillary nerve injury, and the deltoid muscles strength were insufficient. With the functional restoration of axillary nerve after the operation, the signs of shoulder subluxation disappeared at 3 months after the surgery. Therefore, deltoid muscle could be one of the important factors to maintain the stability of the shoulder joint.

Many scholars has found that the indications of young patients with comminuted fractures, If the bone mass in the region of scapula glenoid side were excellent, the procedures of humeral head replacement still exists controversy^9^, ^13^. Therefore, in this study, we enrolled the patients with the combination of comminuted fractures, whose age were larger than 60 years old, and bone mass showed obvious osteoporosis. Through preoperative X‐ray and CT examination, the results showed that humeral head could not be repaired, and we conducted the humeral head replacement as the one‐stage operation for these patients.

**Fig. 3:**
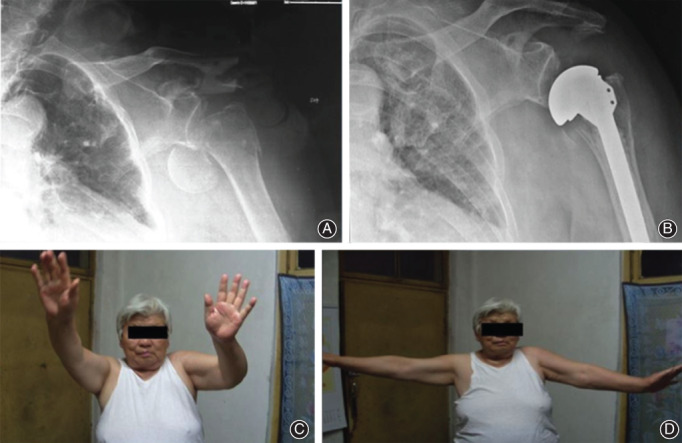
A female, 73 years old, underwent left side shoulder hemiarthroplast for the treatment of left side comminuted proximal humeral fracture. (A, B) indicated the X‐ray outcome of the patient. During the operation, it could be observed rotator cuff tear. (C, D) indicated the postoperative shoulder joint activity in early period was poor, contributing to rotator cuff. The deltoid muscle was atrophic and the artificial femoral head showed subluxation status. After 3 years of follow‐up, the abduction, anteflexion were very poor.

**Fig. 4:**
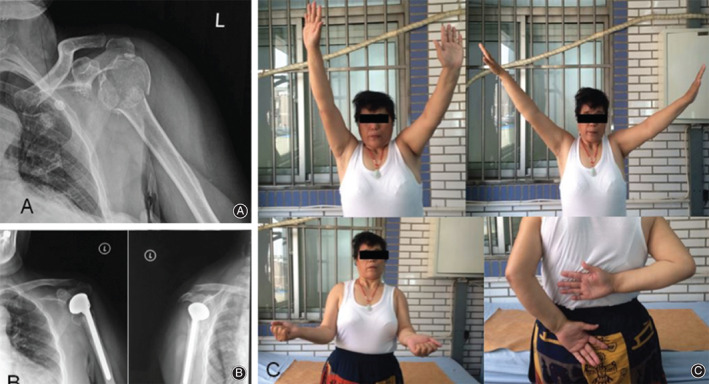
A female, 54 years old, was diagnosed as left side proximal humeral fracture caused by traffic accident. (A, B) indicated that the result of preoperative X ray suggested comminuted proximal humeral fracture according to Neer classification. The patient underwent left side humeral head replacement. (C) indicated that after 5 year follow‐up, prosthesis position was good. Though the part of boss mass in the region of lesser trochanter showed malunion, it did not influence anteflexion, lifting, function of the shoulder joint, and the part function of external rotation and internal rotation were limited in this patient.

The postoperative rehabilitation of artificial humeral head replacement has not been paid enough attention by the surgeons. Although the range of motion in shoulder joint was mainly dependent on the surgical procedure of humeral head replacement, reasonable and early rehabilitation intervention is still very crucial for the recovery of shoulder joint function^18^, ^19^. In a study by Soliman *et al*., the postoperative rehabilitation plan was established from the time point of 4 weeks after the surgery. In the previous study, the humeral shaft brace was applied to fix the shoulder joint in the neutral position, in order to ensure the healing of both bone mass and soft issue^17^. In a study by Gallinet *et al*., the passive shoulder rehabilitation activities was advanced to the immediate postoperative time, and the active shoulder joint activity began at time point of 45 days after operation^14^. Considering the above‐mentioned rehabilitation process of shoulder joint, we used the brace to fix the shoulder joint in the neutral position immediately after the operation. Meanwhile, the patients were required to prohibit any active exercises for 3 weeks after the operation. During the period of 3–4 weeks after the operation, X ray of shoulder joint was routinely reviewed, in order to evaluate whether the position of artificial humeral head was changed or not. If the position of artificial humeral head did not suffer special shift, the active shoulder joint exercise should be conducted under the protection of the rehabilitation technicians. On the premise of the surgical factor excluded, the range of motion in the shoulder joint towards each direction in eight cases of our study were approximately close to that of the contralateral limb (Fig. 3,4).

This research has some limitations. First, this is a retrospective study, we did not get a trial group as contrast. Second, the types of the fracture dislocation include three part or four part fractures, we did not separate the subgroups according to the fracture types. These should be studied further in future research.

### *Conclusion*

The humeral head replacement was still one of the most convenient and feasible methods for the treatment of three part or four part proximal humeral fracture. During the surgery process, the stable structure of shoulder joint could be completely restructured, and the rehabilitation plan should be adjusted reasonably and timely after the operation.
